# Classification of 27 Tumor-Associated Antigens by Histochemical Analysis of 36 Freshly Resected Lung Cancer Tissues

**DOI:** 10.3390/ijms17111862

**Published:** 2016-11-08

**Authors:** Gene Kurosawa, Mototaka Sugiura, Yoshinobu Hattori, Hiroyuki Tsuda, Yoshikazu Kurosawa

**Affiliations:** 1 Division of Antibody Project, Institute for Comprehensive Medical Science, Fujita Health University, Toyoake 470-1192, Japan; gene@fujita-hu.ac.jp; 2 Internal Medicine, School of Medicine, Fujita Health University, Toyoake 470-1192, Japan; msugiura@fujita-hu.ac.jp; 3 Division of Thoracic and Cardiovascular Surgery, School of Medicine, Fujita Health University, Toyoake 470-1192, Japan; y-hattori@daidohp.or.jp; 4 Department of Toxicology, Graduate School of Medical Sciences, Nagoya City University, Nagoya 467-8603, Japan; htsuda@phar.nagoya-cu.ac.jp; 5 Department of Surgery, Social Medical Corporation Kojunkai Daido Hospital, Nagoya 457-8511, Japan

**Keywords:** cancer-associated antigens, histochemical analysis, phage-display antibody library, therapeutic antibody, combination therapy

## Abstract

In previous studies, we identified 29 tumor-associated antigens (TAAs) and isolated 488 human monoclonal antibodies (mAbs) that specifically bind to one of the 29 TAAs. In the present study, we performed histochemical analysis of 36 freshly resected lung cancer tissues by using 60 mAbs against 27 TAAs. Comparison of the staining patterns of tumor cells, bronchial epithelial cells, and normal pulmonary alveolus cells and interalveolar septum allowed us to determine the type and location of cells that express target molecules, as well as the degree of expression. The patterns were classified into 7 categories. While multiple Abs were used against certain TAAs, the differences observed among them should be derived from differences in the binding activity and/or the epitope. Thus, such data indicate the versatility of respective clones as anti-cancer drugs. Although the information obtained was limited to the lung and bronchial tube, bronchial epithelial cells represent normal growing cells, and therefore, the data are informative. The results indicate that 9 of the 27 TAAs are suitable targets for therapeutic Abs. These 9 Ags include EGFR, HER2, TfR, and integrin α6β4. Based on our findings, a pharmaceutical company has started to develop anti-cancer drugs by using Abs to TfR and integrin α6β4. HGFR, PTP-LAR, CD147, CDCP1, and integrin αvβ3 are also appropriate targets for therapeutic purposes.

## 1. Introduction

More than 20 years have passed since the success of trastuzumab against HER2 for the treatment of breast cancer [1]. Although many groups, including large pharmaceutical companies, have attempted to develop therapeutic monoclonal antibodies (mAbs) against solid cancers, the number of successful examples is limited [2,3]. However, in the case of hematological malignancies, more than a dozen mAbs have been approved as therapeutic drugs [2,4]. In these cases, the targets do not have to be tumor-associated antigens (TAAs), because normal cells that express the target molecules are produced from the bone marrow stem cells after the treatment. Moreover, the Abs easily reach the malignant cells to trigger antibody-dependent cell mediated cytotoxicity and complement-dependent cytotoxicity and effectively kill them [5,6]. Recently, however, a new concept in the development of anti-cancer drugs, immune checkpoint blockade, has changed the role of Abs for killing of tumor cells [7]. In the immune system, T-cell activation is highly regulated by immune checkpoint molecules that include cytotoxic T lymphocyte antigen 4 (CTLA-4), programmed cell death protein 1 (PD-1), and ligand for PD-1 (PD-L1). These 3 molecules have been shown to be good targets for cancer therapy [8,9]. If their function is inhibited, cytotoxic T cells that can recognize tumor-specific peptide-bound HLA molecules are activated to kill the cancer cells. Thus, ipilimumab, which blocks CTLA-4 [8], and pembrolizumab and nivolumab, which both block PD-1, have been developed and approved by the FDA [9]. This therapeutic approach, however, does not limit T-cell activation to only cancer cells. When this therapy is successful in killing tumor cells, the tumor cells completely disappear from the patient’s body. Unfortunately, the percentage of patients who respond to this therapy is relatively low [8,9]. Therefore, combination therapies, such as immune checkpoint blockade plus the specific killing of tumor cells, should be developed.

The specific killing of tumor cells by mAbs could still be a hopeful option. In our previous study we used the word TAA as a practically useful meaning as follows. Many human mAbs isolated from the library termed AIMS were individually screened using at least three different fresh tumor tissues. Based on the immunostaining patterns in the histochemical sections they were classified. When mAbs significantly stained only the surface of tumor cells but negatively or very weakly stained the other normal cell, we tentatively judged the target as TAAs. According to the criteria, we identified 29 TAAs and isolated 488 human mAbs that specifically bind to one of the 29 TAAs [10,11]. Therefore, most of the TAAs identified in our study are expressed on normal growing cells at a low level. However, this difference in the expression level of TAAs between normal growing cells and cancer cells could be utilized for preferential killing of tumor cells, therefore, for the development of therapeutic drugs against cancers. Thus, the results from the present study are informative for the selection of proper target molecules for cancer therapies.

## 2. Results

### 2.1. Classification of Staining Patterns

**Figure 1:**
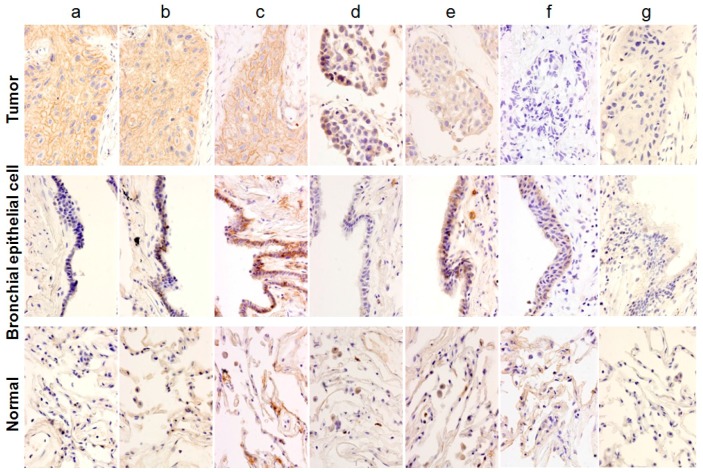
Classification of histochemical analysis. The patterns obtained by the histochemical analysis were classified into 7 categories: (**a**–**g**) The staining patterns of three portions, namely tumor cells, bronchial epithelial cells, and normal pulmonary alveolus cells and interalveolar septum, were compared. The details of classification are described in the text.

A total of 36 fresh lung cancer specimens were analyzed with 60 different mAbs against the 27 TAAs. Cancerous tissues and surrounding cancer-free tissues were separately cut into small pieces and subjected to immunohistochemical (IHC) analysis. The staining patterns of (1) tumor cells; (2) bronchial epithelial cells; and (3) normal pulmonary alveolus cells and interalveolar septum were compared. Individual staining patterns were classified into 7 categories: category *a*, in which the surface of tumor cells was specifically stained; category *b*, in which both the surfaces of tumor cells and bronchial epithelium cells that represent normal growing cells were stained; category *c*, in which the surface of tumor cells, bronchial epithelial cells, and pulmonary alveolus cells were stained; category *d*, in which the cytoplasmic portion of tumor cells was specifically stained; category *e*, in which the cytoplasm of tumor cells and some portions of normal cells in the interstitium were stained; category *f*, in which tumor cells were not stained and some portions of normal cells were stained; and category *g*, in which there was no staining. Representative examples of each category are shown in Figure 1.

### 2.2. Immunohistochemical (IHC) Analyses of 36 Fresh Lung Cancer Specimens with 60 Monoclonal Antibodies (mAbs) against 27 Tumor-Associated Antigens (TAAs)

**Figure 2:**
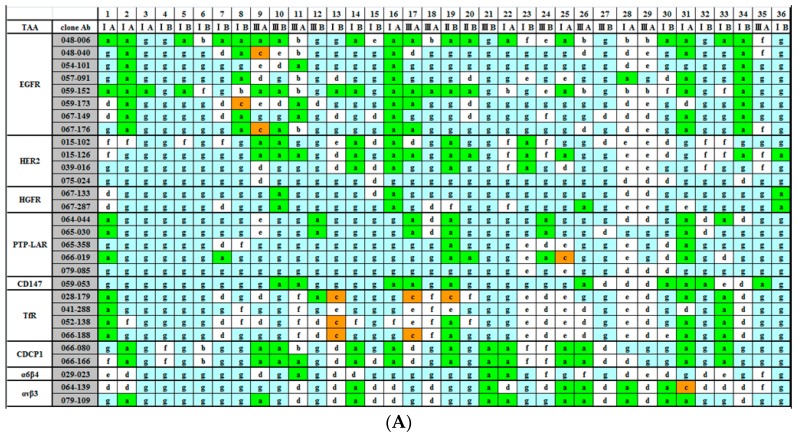

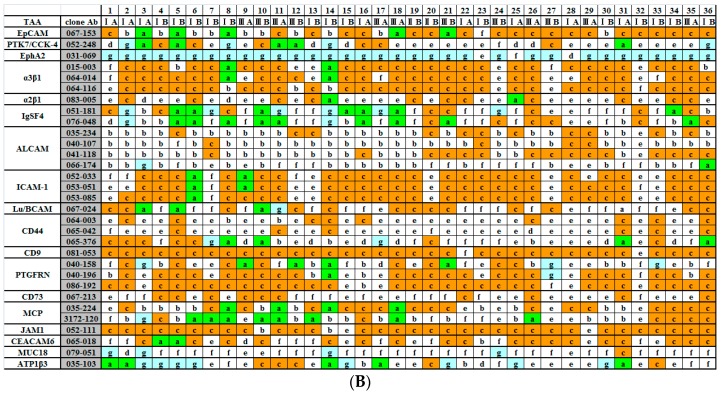
Results of the histochemical analyses. The total of 2160 IHC (immunohistochemical) patterns were classified into 1 of 7 categories. The definition of respective category is described in the text and the examples of IHC patterns are shown in Figure 1. Category *a*, *c* and *g* are marked in green, orange and blue, respectively. (**A**) The results with Abs (antibodies) against 9 TAAs (tumor-associated antigens) indicated that these Abs could be candidates as therapeutic drugs, since these Ags could be considered as authentic TAAs; (**B**) The results with Abs against 18 Ags indicated that these Ags could not be considered as TAAs.

A total of 2160 IHC analyses were performed. The results are shown in Figure 2A,B. Since three types of expression are critical for judgement, i.e., category *a* indicates tumor-cell surface-specific expression, category *c* indicates both tumor cell surface and normal cell surface expression, and category *g* indicates no expression in any cell type, they are marked in green, orange, and blue, respectively. Since the majority of patterns for 9 TAAs are classified into category *a* or *g*, they could be considered to be authentic TAAs, and therefore are shown together in Figure 2A. Multiple Abs against EGFR and HER2 were utilized for staining. In cases where Abs that bound to the same molecule gave different results, these differences should be derived from the differences in the binding activity and/or the epitope. While 048-006 and 059-152 seemed to be promising candidates as anti-cancer drugs from our data, we have already reported that 048-006 and 059-152 caused different effects on cancer cells in a previous paper [10].

For the other 18 TAAs, the majority of the patterns were categorized as type *b*, *c*, *e*, or *f*, except for EphA2. For EphA2, there was no category *a*, that is, no case where it was clearly expressed on the surface of tumor cells. Integrin α3β1, ICAM1, CD9, PTGFRN, and JAM1 were mainly classified as type *c*. ALCAM was type *b*, CD44 was type *e*, and MUC18 was type *f*. EpCAM was a mixture of types *b* and *c*, while integrin α2β1 was a mixture of types *c* and *e*. For IGSF4, one third of the staining was category *a*. The other 6 Ags, PTK7, Lu/BCAM, CD73, MCP, CEACAM6, and ATP1β3, gave more complicated patterns. Type *b* indicates that the Ag plays a role in normal growing cells. In the case of type *c*, the Ag plays a role even in normal quiescent cells. Although all of the TAAs identified according to our experimental strategy should play some role in tumorigenesis, we do not think that these 17 TAAs, with the possible exception of IGSF4, are good candidates as drug targets, and therefore are shown together in Figure 2B.

## 3. Discussion

Our strategy for the development of therapeutic Abs against solid cancers is as follows. As the first step, we isolated a large number of human mAbs that bind to the surface of tumor cells by using a phage-display human Ab library. They were individually screened by immunostaining, and clones that preferentially and strongly stained the malignant cells were chosen. The Ags recognized by those clones were isolated by immunoprecipitation and identified by mass spectrometry. Thus, we successfully identified 29 TAAs and isolated 488 human mAbs that specifically bind to 1 of the 29 TAAs [10,11]. In the present study, we performed an extensive IHC analysis with a large number of lung cancer tissues. We have previously performed similar experiments using hepatocarcinoma, gastric cancer, pancreatic carcinoma, renal carcinoma, and prostate cancer. We found that CEACAM6, JAM1, and integrin α3β1 are expressed at high levels and high frequencies in pancreatic carcinomas. A high level of EpCAM expression is prominent in gastric cancer, and a high level of IGSF4 expression is frequently observed in hepatocarcinomas. In the present study, however, the results using Abs against CEACAM6, JAM1, integrin α3β1, and EpCAM indicated that more than half of the IHC patterns were categorized as type *c*, which means that these proteins are frequently expressed at a high level in normal growing cells and even in normal resting cells. Among them EpCAM has been historically one of the most famous Ags as a target for therapeutic Abs against cancers and several groups are still trying to develop them although the form of Abs has been changed from a simple mAb [12]. For IGSF4, one third of the IHC patterns were type *a*, but the remaining patterns were either type *c* or *f*. Therefore, we did not think that these molecules are good targets for therapy. PSMA is the only TAA with high levels of cell surface expression on 100% of cancer cells but 0% of normal cells [13]. We wonder if there are any other TAAs that are specifically utilized in the tumorigenesis of a specific type of cancer. Even for PSMA, it is not known how this antigen is involved in the tumorigenesis of prostate cancer. When we tried to find a relationship between the type of cancers used for screenings and the kind of TAAs identified, it was very difficult to find cases in which Abs against some specific TAAs had been isolated only from screenings with a specific type of cancer cell. For example, Table 1 in our previous paper [11] indicated how 488 kinds of mAbs had been isolated. It was impossible to find a correlation between the type of cancer cell used for screening and the kind of TAAs against which mAbs had been isolated. In the case of TAAs against which many kinds of Abs were isolated, they were expressed at high levels on the surface of many types of cancer cells. This could be a general phenomenon observed in cancer cells. Therefore, we argue that although only lung cancer tissues were analyzed in the present study, the observations obtained in the present study might be applied to many kinds of solid cancers. Therefore, the most important conclusion in the present study is that 9 TAAs listed in Figure 2A could be good targets for therapeutic Abs against various solid tumors.

EGFR and HER2 are the only TAAs against which mAbs have been successfully developed as therapeutic drugs against solid cancers [3]. One of the reasons why EGFR and HER2 are good targets could be that the signals through EGFR and HER2 are indispensable for growth of tumor cells. The data shown in Figure 2A indicate that even simultaneous expression of both EGFR and HER2 at high levels occurred at a certain frequency. Although the development of anti-HGFR Abs as anti-cancer drugs has been tried for many years [14], there has been no success. For cell survival of tumor cells, the signal pathway through HGFR could be different from EGFR and HER2 pathways in terms of indispensability. PTP-LAR is a receptor-type tyrosine phosphatase [15]. While the human genome encodes more than 20 kinds of receptor-type tyrosine phosphatases, only PTP-LAR is known to be overexpressed in tumor cells [16]. While the growth signal through phosphorylation of tyrosine residues on growth factor receptors such as EGFR appeared to be stopped by de-phosphorylation with a receptor-type tyrosine phosphatase, there has been no good explanation for the frequent overexpression of PTP-LAR in cancer cells. In any case, our present data suggest the possibility of PTP-LAR as a target for therapeutic Abs.

Abs against 2 Ags, TfR and integrin α6β4, among the 9 TAAs listed in Figure 2A are being used by a pharmaceutical company to develop anti-cancer drugs [17,18]. There are more than 20 kinds of integrin. Although high-level expression of some of them (for example, integrin α3β1 and integrin α2β1) in tumor cells has been reported, they are also expressed at high levels in normal cells, as shown in Figure 2B. Among them, therefore, integrin α6β4 and αvβ3 could be candidates as indicated in Figure 2A [18,19]. CDCP1 is an anoikis regulator [20], and a couple of groups have been trying to develop anti-CDCP1 Ab for cancer therapy [21]. The reason why CD147, a metalloproteinase inducer, is overexpressed in various types of tumors is not well understood. An anti-CD147 human-mouse chimeric Ab (metuzumab) has been developed for the treatment of non-small cell lung cancer by a Chinese group [22].

In this experiment we used multiple Abs against certain TAAs. For example, eight mAbs were utilized against EGFR. In a separate experiment we examined the effects of these Abs on the growth of tumor cells expressing EGFR In Vitro and In Vivo, that is, in athymic mice and reported that the results are very heterogeneous among the Abs that included already approved therapeutic Abs, Erbitux and Vectibix [23]. Differences observed among the clones against the same Ag in Figure 2 could reflect this heterogeneity.

## 4. Materials and Methods

### 4.1. mAbs

A total of 60 mAbs against 27 different TAAs were used for histochemical analysis. All of them were previously isolated by our group as previously described [10,11]. The single-chain Fv (scFv) form of Ab linked to a truncated cp3 was used for histochemical analyses.

### 4.2. Immunostaining of Lung Cancer Tissues

Surgically resected tumor tissues were immunostained according to the previously published procedure [24]. The freshly resected lung cancer tissues, including the cancer-free surrounding areas that contained bronchial epithelial cells and normal pulmonary alveolus cells and inrteralveolar septum, were further cut into small blocks (5 mm × 5 mm × 5 mm), placed in 4% paraformaldehyde/0.01% glutaraldehyde in 0.1 M cacodylate buffer (pH 7.4) at 4 °C, and treated in a microwave oven for 30 s. Then, it was fixed again in the above fixation solution at 4 °C for 1 h, then substituted by 15% sucrose/PBS and immersed therein at 4 °C for 4 h, and then substituted by 20% sucrose/PBS and immersed overnight. It was embedded in OCT (Optimal Cutting Temperature) compound (Sakura Finetek Japan Co., Ltd., Tokyo, Japan) and rapidly frozen in dry ice. Frozen specimens were cut into 3–5 μm thickness by using cryostat, attached to silane coated slide glass and dried by using a cold wind dryer for 30 min. They were stocked at −80 °C. After the slide to which a section was attached was immersed in PBS, 50 μL of 0.3% H_2_O_2_/0.1% NaN_3_ was dropped, washed with PBS. blocked with 2% BSA/PBS, reacted with mAb which was in the form of scFv fused with truncated cp3; next, the sections were stained with rabbit anti-cp3 antiserum followed by goat anti rabbit IgG antiserum conjugate with HRP.

### 4.3. Ethics Statement

This project received the permission from the ethical committee in Fujita Health University and all the patients provided written informed consent for usage of removed cancer tissues in this study. Approval was issued in July 2005 (identification code: 05-016) by the Ethics Review Committee organized by president of Fujita Health University.

## 5. Conclusions

Among the 27 TAAs that had been identified in our previous studies we judged the following 9 Ags to be proper targets for therapeutic Abs against the solid cancers: EGFR, HER2, HGFR, PTP-LAR, TfR, integrin α6β4, integrin αvβ3, CD147 and CDCP1.
